# Liquid Hydrolyzed Fish Protein (Anchovy) in the Canine Diet: A Focus on Gut Fermentation and Fecal Quality

**DOI:** 10.3390/vetsci12080779

**Published:** 2025-08-20

**Authors:** Elisa Martello, Annalisa Costale, Fabrizio Ferrarini, Diana Vergnano, Gianandrea Guidetti, Giorgia Meineri

**Affiliations:** 1 Centre for Evidence Based Healthcare, School of Medicine, University of Nottingham, Nottingham NG5 1PB, UK; 2 Department of Drug Science and Technology, University of Turin, 10125 Turin, Italy; annalisa.costale@unito.it; 3 FarPro Modena S.P.A., 41057 Spilamberto, Italy; fabrizio.ferrarini@farpro.it; 4 About Petfood, 10048 Vinovo, Italy; diana@aboutpetfood.it; 5 SANYpet S.P.A., 35023 Bagnoli di Sopra, Italy; gianandrea.guidetti@nastapetfood.com; 6 Department of Veterinary Sciences, School of Agriculture and Veterinary Medicine, University of Turin, 10095 Turin, Italy; giorgia.meineri@unito.it

**Keywords:** digestive health, fecal metabolites, gut health, hydrolyzed food, putrefactive

## Abstract

Hydrolyzed protein is increasingly used in pet food, especially for animals with adverse reactions to food or gastrointestinal issues. This randomized, double-blind controlled trial evaluated the effects of a diet containing hydrolyzed anchovy protein on the gut health of healthy adult West Highland white terriers (5 males, 25 females; 5.6–9 kg). The dogs were randomly assigned to a control group (CTR, *n* = 15), receiving a standard commercial diet, or a treatment group (TRT, *n* = 15), fed a diet partially replacing anchovy meal with hydrolyzed anchovy protein for 42 days. All the dogs underwent a veterinary health check at baseline (T0) and remained healthy throughout the study. The food and water intake were recorded daily. The body weight (BW), body condition score (BCS, 1–9), and fecal score (FS, 1–7) were assessed at days 0, 21, and 42. Fecal samples were collected at T0 and T42 to measure calprotectin, cortisol, and putrefactive compounds (indole and skatole). No significant differences were found between the groups in the BW, BCS, FS, calprotectin, or cortisol. However, the TRT group showed a significant reduction in fecal indole and skatole. The results suggest that hydrolyzed anchovy protein may improve gut fermentation and support better digestive health in dogs, leading to reduced fecal odor and increased owner satisfaction.

## Simple Summary

This study tested a new dog food containing hydrolyzed anchovy protein to see if it improves gut health. The trial involved 30 healthy West Highland white terriers who were split into two groups: One group received a regular commercial diet (control group) and the other received the new diet with hydrolyzed anchovy protein for 42 days. All the dogs had their health checked at the start of the study and remained healthy throughout. The researchers monitored their weight, body condition, and digestive health. The results showed no major changes in weight, body condition, inflammation, or stress markers in the two groups. However, the dogs on the new diet had significantly lower levels of putrefactive compounds (indole and skatole) in their feces. This suggests that a diet with hydrolyzed protein may support healthy gut fermentation and alleviate digestive discomfort, leading to reduced fecal odor and increased owner satisfaction.

## 1. Introduction

Hydrolyzed protein has gained significant attention in the pet food industry, particularly in formulations designed for pets with adverse food reactions (such as allergies) or gastrointestinal disorders [1,2].

The primary goal of protein hydrolysis in specialized diets is to sufficiently alter the protein structure to help prevent immune recognition in individuals already sensitized to the intact protein. Additionally, the hydrolysis process must break down proteins to a level where no antigenic components remain that could trigger an immune response or lead to sensitization in an individual with no prior exposure [3]. Protein hydrolysates are generally expected to have a higher digestibility than their intact protein counterparts. Extensively hydrolyzed proteins in particular appear to serve as an optimal amino acid source, facilitating maximal digestion and absorption [3]. Improved digestion helps maintain optimal nutrient absorption, ensuring that pets receive the essential amino acids needed for growth, maintenance, and overall health. For example, clinical improvement, along with histopathological recovery, has been documented in dogs with refractory inflammatory bowel disease (IBD) when fed a soy-based hydrolyzed diet as the sole treatment [4]. Another study found that a dietary modification with hydrolyzed fish proteins improved gastrointestinal symptoms in non-PLE (nonprotein-losing chronic enteropathies—CE) dogs, aligning with previous reports of long-term clinical remission in dogs with CE [5].

As pet owners increasingly seek high-quality nutrition to support their pets’ health, the inclusion of hydrolyzed proteins in commercial pet food has become a key innovation. One of the most notable advantages of hydrolyzed protein-based diets is their positive impact on gut health, which is essential for overall well-being in pets.

This randomized, double-blinded controlled trial aimed to investigate the effects of replacing part of the anchovy fish protein in a commercial diet with hydrolyzed anchovy fish protein on the nutritional and fecal parameters of healthy dogs.

## 2. Materials and Methods

### 2.1. Animals and Study Design

Thirty adult West Highland white terriers (5 males and 25 females, between 2 and 6 years old, weighing between 5.6 and 9 kg) were chosen from an ENCI (Ente Nazionale Cinofilia Italiana) breeder (La Rossella, Turin, Italy).

This breeder provided written informed consent for their participation in the study. The trial was performed following the guidelines set by the Italian Ministry of Health for the animals’ welfare and received institutional approval from the Ethical Animal Care and Use Committee of the University of Turin (protocol 0031/DSV/UNITO, 4 May 2025).

This study was designed as a randomized, double-blind controlled trial. The dogs were housed in rooms, with two dogs per unit. Each room covered an area of 12 square meters indoors and 26 square meters outdoors, ensuring adherence to animal welfare standards and minimizing social stress due to collective handling.

At the baseline (T0), a veterinarian conducted a general health examination on all the dogs to confirm the absence of any pre-existing medical conditions.

Two groups were formed by randomly assigning the dogs: one control group (CTR, 15) and one treatment group (TRT, 15).

### 2.2. Diet

Both the CTR and TRT groups were fed a commercial diet (control diet, Table 1 and Table 2) for a pre-trial period of seven days. Then, during the 42-day trial period, the CTR group continued to receive the commercial diet, while the treatment group was fed a newly formulated diet with part of the anchovy meal replaced with hydrolyzed anchovy protein (hydrolyzed diet, Table 2). Both diets had the same analytical constituents (see Table 1) and contained the same ingredients except for the addition of hydrolyzed anchovy and the replacement of monocalcium phosphate with calcium carbonate in the hydrolyzed diet. The hydrolyzed anchovy fish protein (average molecular weight <5 kDa) [6] was made with enzymatically hydrolyzed anchovy fish emulsion, stabilized until reaching a pH of 3.5 and containing natural antioxidants.

The daily intake for both diets was set according to the individual diet requirements for adult dogs.

### 2.3. Nutritional Parameters

The veterinarian recorded the body weight (BW) at the baseline (T0) and then after 21 (T21) and 42(T42) days of the trial. The animals were examined by the same trained veterinarian at the three time points to record the body condition score (BCS-score 1–9), with a score of 4 or 5 being the desired outcome [7]. The feed intake and water intake for each animal were registered daily by the breeder.

### 2.4. Fecal Parameters

The fecal score (ranging between 1 and 7, [7]) was recorded by the veterinarian at T0, T21, and T42.

The calprotectin, cortisol, and putrefactive fecal compound (indole and skatole) concentrations were measured using fresh feces collected at T0 and T42.

Fresh feces were collected with a spatula and placed in a sterile plastic bag with a unique code. The samples were kept at 4 °C and then delivered to the laboratory (University of Turin, Italy). The analysis was performed by the same laboratory technician with a blinded protocol.

An enzyme-linked immunosorbent assay (ELISA) was used to measure the fecal calprotectin concentration. An enzyme immunoassay (EIA) was used to measure the cortisol. Gas chromatography was used to determine the concentrations of indole and skatole. Details of the laboratory techniques used in this study are reported in the recently published paper by Atuahene and colleagues (2023) [8].

### 2.5. Statistical Analysis

The statistical analysis used in this study varied according to the type of parameter (e.g., categorical or numerical). Numerical parameters, including the body weight (BW), calprotectin, indole, skatole, and cortisol, were analyzed using an analysis of variance (ANOVA) based on a repeated measures model, implemented via the MIXED procedure (PROC MIXED MODEL SAS 9.4, 2013).

The statistical model was as follows: y = μ + Si + Gj + Tk + GTjk + ejkn, where

- y = dependent variable;
- μ = overall mean;
- Si = fixed effect of sex (i = F; M);
- Gj = fixed effect of treatment (j = 0, 1);
- Tk = fixed effect of the kth time point (k = 1, 6);
- GTjk = fixed effect of the interaction between the jth treatment and the kth time point;
- ejkn = error.

Time was treated as a repeated measure, with the groups considered repeated subjects. An autoregressive covariance structure was applied. The least-squares means were compared using Student’s *t*-test.

The categorical parameters, BCS and FS, for the TRT and CTR groups were compared over the entire experimental period using the Kruskal–Wallis test (UCLA), via the PROC NPAR1WAY procedure in SAS 9.4. When significant differences were detected, multiple comparisons were conducted using pairwise two-sample Wilcoxon tests.

All tests were two-tailed, and results with *p-*values < 0.10 were considered statistically significant.

## 3. Results and Discussion

All the dogs participating in the trial were initially healthy based on a veterinary assessment; they remained healthy throughout the study and did not require any medications at any point. Additionally, they had not undergone any treatment or supplementation in the 15 days preceding the study’s commencement. No significant alterations in their diet consumption or water intake were observed by the breeder for the entire duration of the trial.

At the beginning of the study (T0), none of the tested parameters showed any significant differences between the CTR and TRT groups.

The effects of the diets on the BW, BCS, and FS are presented in Table 3. As expected in a trial involving healthy dogs, the BW and BCS showed no statistically significant differences between the groups at any time point. This finding supports the equivalent energy provision of both diets.

The FS showed no statistically significant differences between the groups at any time point. This result confirms that both diets maintained the fecal appearance and that the new diet did not negatively affect the fecal consistency.

The effects of the new diet on the calprotectin, cortisol, and putrefactive fecal compound (indole and skatole) parameters are presented in Table 4.

The calprotectin and cortisol levels in the dogs did not differ significantly between the groups at any time point. This highlights that there was no clear effect of the new diet on the biomarkers of intestinal inflammation (calprotectin level), stress responses, or immune function (cortisol level) in healthy dogs [9,10]. This result could be linked to the absence of intestinal inflammation and stress status during the study period.

Intestinal bacteria contribute to the production of putrefactive compounds [11], as well as hydrolytic and reductive enzymes, which can negatively affect intestinal health. Tryptophan-derived metabolites, such as indole and skatole, are signaling molecules and play crucial roles in immune regulation (e.g., modulation of T-cell activity), anti-inflammatory processes, and the maintenance of gut barrier integrity [12]. However, the effects of these compounds are highly variable and dose-dependent. When present in excess, substances like ammonia, hydrogen sulfide, indole, and skatole may contribute to adverse outcomes by damaging the intestinal epithelium [13]. Furthermore, these putrefactive compounds are largely responsible for the characteristic odor of feces [14]. In this study, the significant decrease in putrefactive fecal compounds (indole and skatole) in the TRT group suggests that the new diet with the hydrolyzed protein may have had a beneficial effect on gut fermentation, potentially reducing the production of these compounds associated with intestinal discomfort and odor. In contrast, the lack of a significant change in the CTR group indicates that the control diet did not influence the fecal composition in the same way, highlighting the potential efficacy of the hydrolyzed proteins in improving digestive health. In agreement with our findings, a study in dogs showed that putrefactive compounds, fecal isovalerate and total phenol/indole, were lower in dogs fed a diet containing chicken liver together with heart hydrolysate versus a chicken-meal-based diet [15].

A future trial would benefit from a longer duration of diet administration, a larger sample size, and the inclusion of hematological marker evaluations.

## 4. Conclusions

In conclusion, this trial suggests that a diet containing hydrolyzed anchovy fish protein (TRT group) may improve gut fermentation by reducing putrefactive fecal compounds like indole and skatole, potentially enhancing digestive health and reducing intestinal discomfort. The control diet showed no significant changes, indicating that a hydrolyzed protein diet may have specific benefits. However, no clear effects were observed on the biomarkers of inflammation or stress, highlighting the diet’s influence mainly on fecal composition rather than on systemic health markers. This could result in a positive impact for dog owners, as it may help reduce unpleasant fecal odor, and also for the dogs themselves, by improving their overall gastrointestinal health.

## Figures and Tables

**Table 1:** Analytical constituents of both control and hydrolyzed diet.

Analytical Constituents	Unit	Hydrolyzed Diet	Control Diet
Moisture	%	8.03	8.02
Crude protein	%	32.41	32.55
Crude fat	%	17.1	17.07
Crude ash	%	7.36	7.3
Crude fiber	%	2.37	1.66

**Table 2:** Hydrolyzed and control diet compositions.

Ingredients	Hydrolyzed Diet (%)	Control Diet (%)
Anchovy meal	19.08	28.4
Potato starch	5.46	15.03
Dried peas	18.07	10.02
Chicken fat	5.72	6.01
Salmon oil	5.02	6.01
Monocalcium phosphate	-	1.7
Liquid palatant	1.5	1.5
Ascophyllum nodosum	1	1
Vitamin mineral premix	0.52	0.52
Dry palatant	0.2	0.2
Taurine	0.1	0.1
Dried cranberries	0.04	0.04
Hydrolyzed anchovy	13.05	-
Calcium carbonate	0.9	-

**Table 3:** Body weight (BW), body condition score (BCS), and fecal score (FS) parameters measured in the treated (TRT) versus untreated (CTR) dogs at different time points (T0, T21, and T42).

	T0	T21	T42	*p-*Value *
**BW (±SE) (Kg)**				
CTR	6.9 ± 0.2	7.1 ± 0.2	7.3 ± 0.2	0.4
TRT	7.3 ± 0.2	7.3 ± 0.2	7.2 ± 0.2	0.9
**BCS (±SE) (1–9)**				
CTR	4.1 ± 0.1	4.1 ± 0.1	4.2 ± 0.1	0.7
TRT	4.3 ± 0.1	4.3 ± 0.1	4.2 ± 0.2	0.9
**FS (±SE) (1–7)**				
CTR	2.5 ± 0.0	2.5 ± 0.0	2.6 ± 0.0	0.4
TRT	2.5 ± 0.0	2.6 ± 0.0	2.5 ± 0.0	0.4

* Significance set at *p* < 0.10.

**Table 4:** Calprotectin, cortisol, and putrefactive fecal compound (indole and skatole) parameters measured in the treated (TRT) versus untreated (CTR) dogs at different time points.

	T0	T42	*p-*Value *
**Calprotectin (±SE) (μg/g)**			
CTR	6.7 ± 0.1	6.5 ± 0.1	0.3
TRT	6.4 ± 0.1	6.5 ± 0.1	0.6
**Cortisol (±SE) (pg/mg)**			
CTR	0.6 ± 0.0	0.6 ± 0.0	0.8
TRT	0.6 ± 0.0	0.6 ± 0.0	0.4
**Indole (±SE) (μmol/g)**			
CTR	3.1 ± 0.1	3.1 ± 0.1	0.8
TRT	3.1 ± 0.1	1.3 ± 0.2	<0.0001
**Skatole (±SE) (μmol/g)**			
CTR	3.7 ± 0.1	3.7 ± 0.1	0.8
TRT	3.7 ± 0.1	2.0 ± 0.1	<0.0001

* Significance set at *p* < 0.10.

## Data Availability

The data that support the findings of this study are available from the corresponding author (E.M.) upon request.
